# Vision-based estimation of manipulation forces by deep learning of laparoscopic surgical images obtained in a porcine excised kidney experiment

**DOI:** 10.1038/s41598-024-60574-w

**Published:** 2024-04-27

**Authors:** Kimihiko Masui, Naoto Kume, Megumi Nakao, Toshihiro Magaribuchi, Akihiro Hamada, Takashi Kobayashi, Atsuro Sawada

**Affiliations:** 1 Department of Urology, Graduate School of Medicine, Kyoto University, 54 Shogoin-kawahara-cho, Sakyo-ku, Kyoto, 606-8507 Japan; 2 Department of Medical Informatics, Graduate School of Medicine, Kyoto University, Kyoto, Japan; 3 Department of Advanced Medical Engineering and Intelligence, Graduate School of Medicine and Faculty of Medicine, Kyoto University, Kyoto, Japan; 4 Department of Urology, Faculty of Medicine, University of Miyazaki, 5200 Kiyotakecho Kihara, Miyazaki, 889-1692 Japan

**Keywords:** Urology, Biomedical engineering, Imaging

## Abstract

In robot-assisted surgery, in which haptics should be absent, surgeons experience haptics-like sensations as “pseudo-haptic feedback”. As surgeons who routinely perform robot-assisted laparoscopic surgery, we wondered if we could make these “pseudo-haptics” explicit to surgeons. Therefore, we created a simulation model that estimates manipulation forces using only visual images in surgery. This study aimed to achieve vision-based estimations of the magnitude of forces during forceps manipulation of organs. We also attempted to detect over-force, exceeding the threshold of safe manipulation. We created a sensor forceps that can detect precise pressure at the tips with three vectors. Using an endoscopic system that is used in actual surgery, images of the manipulation of excised pig kidneys were recorded with synchronized force data. A force estimation model was then created using deep learning. Effective detection of over-force was achieved if the region of the visual images was restricted by the region of interest around the tips of the forceps. In this paper, we emphasize the importance of limiting the region of interest in vision-based force estimation tasks.

## Introduction

Recently, many surgeries have shifted from open to laparoscopic surgery and robot-assisted laparoscopic surgery. Additionally, more minimally invasive surgeries are being performed. Laparoscopic surgery, including robot-assisted surgery, results in shorter length of hospital stay because of smaller wounds and less blood loss compared with open surgery. However, while open surgery involves hand manipulation of organs, laparoscopic surgery involves manipulation using forceps. In laparoscopic surgery, attenuation of haptics is a problem, especially for surgical trainees. Additionally, haptics are impossible for surgeons to detect in robot-assisted surgery, and failure to understand this characteristic may result in intraoperative organ damage and unnecessary blood loss. Enayati et al. stated that “the absence of haptic feedback in minimally invasive surgery can be a cause of error” and reported that most intraoperative organ injuries by surgical trainees were caused by applying more force beyond the safety margin^1^.

There are three main steps in surgical technique training: (1) During open surgery, surgeons can learn with the naked eye the flexibility and elasticity of each organ and tissue, as well as the fragility of the tissue using their hands. (2) Non-robot-assisted laparoscopic surgery confirms the flexibility and elasticity of the tissue through the forceps, and the degree of deformation of the tissue is visible on the image. (3) On the basis of experience with (1) and (2), robot-assisted laparoscopic surgery can be performed safely and minimally invasively without applying unnecessary pressure or force to the tissues beyond the safety margin, even in situations of no sense of touch. However, with the widespread adoption of robot-assisted surgery, there has been an increase in instances where training in surgical techniques commences directly with step (3), omitting preliminary steps (1) and (2). As a result, there is concern about the assurance of surgical safety. To overcome the problem of minimal or absent haptic sensation, biomedical engineering has focused on the use of tactile sensors on the tips of laparoscopic forceps or robotic arms. However, most of these systems are independent systems^2,3^, and only a few have been introduced in clinical practice^4^, limiting the environment and conditions under which haptic sensation can be felt and used. Furthermore, even if new forceps equipped with a force sensor were developed, the challenge of inserting the forceps into the human body necessitates that the forceps meet advanced standards of cleanliness and safety as a medical device. Creating such a device at a production cost that is deemed acceptable presents an exceedingly high hurdle.

Lecuyer et al. presented the concept of “pseudo-haptic feedback” in 2000, and reported the phenomenon in which vision influences haptic perception^5,6^. According to our observations, surgeons who have obtained laparoscopic surgery skills, which involve minimal haptic feedback, tend to sense haptics-similar sensations during the initial stages of robot-assisted laparoscopic surgery, which does not involve haptics. This sensation is considered pseudo-haptic feedback. Furthermore, instructor surgeon can assess the magnitude of force applied to organs from the surgery video images, alone.

As surgeons who routinely perform open, non-robotic, and robot-assisted laparoscopic surgery, we address the issue of how to make haptics-similar sensations explicit to trainee surgeons. Hence, this study applied the concept of “vision-based sensing of forces” reported by Wang et al.^7^, to the surgical environment. Previous studies mainly focused on pushing maneuvers using artificial organ models. Some studies have reported force estimation with multiple camera images taken from varying angles^8^. Other reports have explored force estimation by reconstructing images using three-dimensional (3D) computer graphics^9^ and binocular stereo cameras^10^.

Overall, while some promising advancements have been made in the haptics domain, further research is necessary to apply these techniques in real-world settings. Many previous studies have not collected data from actual surgical procedures; therefore, using the models in these studies in actual surgical environments is not ideal. In this study, we aimed to achieve vision-based estimations of the force magnitudes during forceps organ manipulation, especially during pushing, using images from manipulation records. We also attempted to detect over-force, which exceeds the threshold of safe manipulation. Using an endoscopic system designed for use in actual clinical surgery, images of manipulated excised pig kidneys were recorded with synchronized force data. An estimation model was developed using a convolutional neural network (CNN)^11^ with VGG-16 as a deep learning method.

Specifically, we focused on the importance of limiting the region of interest around the tips of the forceps in vision-based force estimation tasks. This finding is based on our experimental results, which are discussed in detail in the following section. The proposed estimation model requires input images, which represent manipulation in a laparoscopic surgical environment, with synchronized force data for learning. To observe the estimated intention area in the images, a saliency map^12^ was superimposed on the manipulation images. The learning procedure and evaluation methods were optimized by observing the estimated intention area.

## Results

### Experimental setup

**Figure 1:**
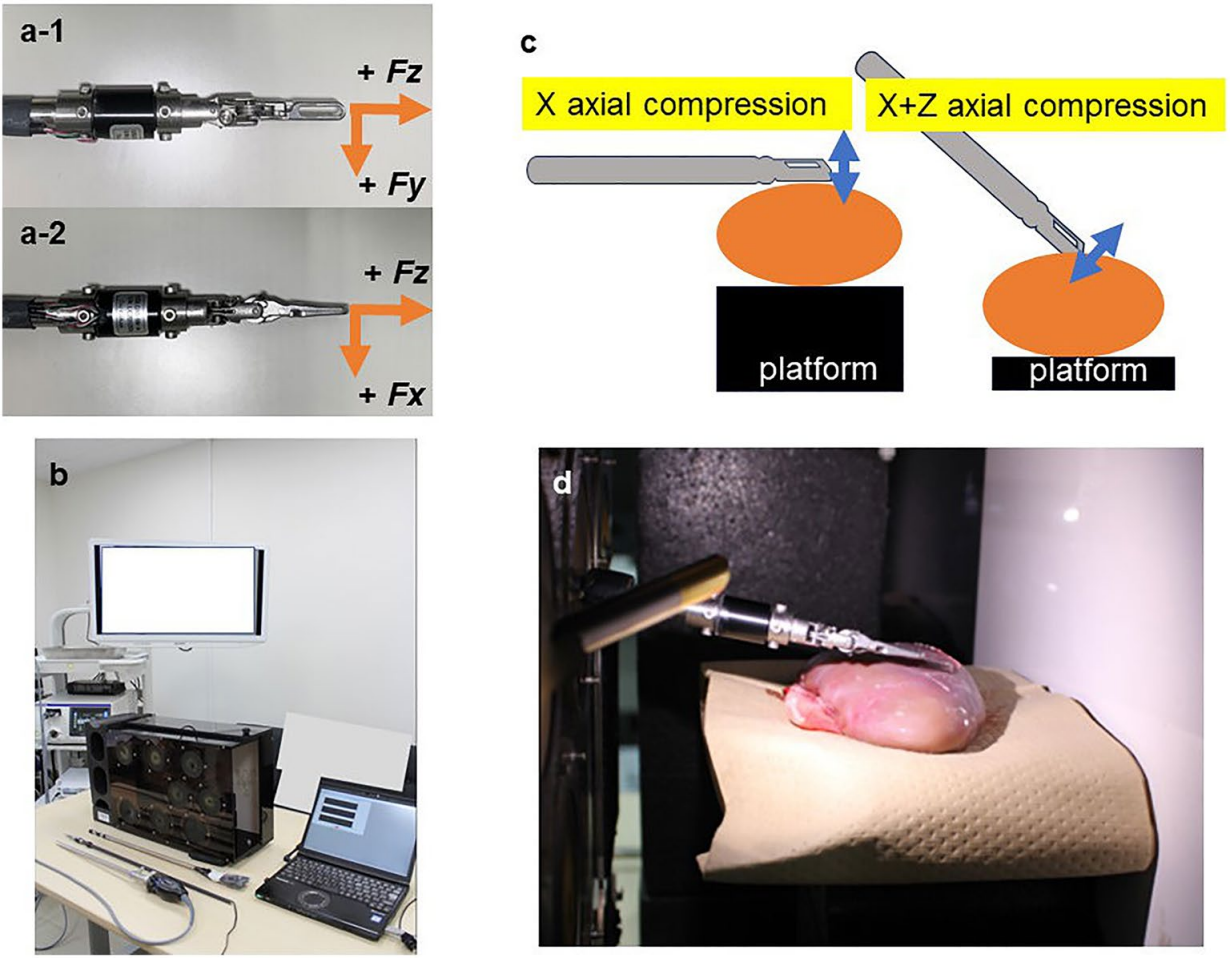
Experimental environment and forceps with a three-axis force sensor. (**a**) Forceps with the three-axis force sensor. (**b**) Box trainer, laparoscopic surgical system, and power measuring systems. (**c**) Experimental conditions for organ compression from two directions. () indicates the direction of movement of the forceps. () indicates excised porcine kidney. (**d**) Inside the box trainer during measurement.

We developed a sensor attached to forceps equipped with a three-axis force sensor based on the forceps tips of a surgical robot (Fig. 1a). Time series force data, which were measured during pressing manipulation with the forceps using excised pig kidneys, were recorded with the visual images of the deformation. A deep learning model was developed that estimated the force on the tips of the forceps using visual images of the organ deformation. During the experiment, we measured the force generated at the tips of the forceps when the forceps were used to press an excised pig kidney. The recorded force and visual images were applied to the deep learning model to optimize the estimation. In the experiments, we used a box trainer (Fig. 1b), which is used in training for laparoscopic surgery. The objects to be pressed by the forceps in the box trainer were the excised kidneys of three pigs. The experimental environmental conditions were a temperature of 24 °C and humidity of 58%. To evaluate the model, three main experimental conditions were set, and two sub-conditions were given for each condition. The main conditions were as follows: (i) level of the force, (ii) direction of the force, and (iii) position of the force when the forceps pressed the organ. The secondary conditions were as follows: (a) Level 1: force applied in a normal laparoscopic operation, Level 2: strong force considered dangerous in a normal operation, (b) X-axis direction and X + Z axes directions of the manipulation force, and (c) front end and center of the organ. Combinations of these conditions were defined as a measurement unit; i.e., a sequence. There were eight sequences for each individual organ measurement. For example, sequence 1: force level 1-direction x-axial pressure position organ center; sequence 2: force level 2-direction x-axial pressure position organ center, and so on. Regarding (ii) (X- and X + Z-axes directions), we confirmed that the force was generated with a uniform motion by changing the position of the organs, as shown in Fig. 1c in terms of the height of the forceps and the angle at which pressure was applied. Before all measurements, calibration of the sensor was performed by measuring the force of a 100-g weight. In the recording environment in the box trainer, the VISERA ELITE II system (Olympus Co., Tokyo, Japan) was used as the endoscopic system, and the WA4KL130 (Olympus Co.) endoscope with a 10-mm diameter high-resolution optical channel was used for video recording (Fig. 1b, d). The video recording was obtained using monocular two-dimensional (2D) endoscopic images. The dataset learning was also performed using 2D images.

### Training and testing

We matched the force generated at the forceps tips when an organ was pressed (forceps pressure) over time with video recordings of organ deformation over time when the organ was pressed using the forceps. Then, each sequence was analyzed using a CNN. The model was evaluated using three setting steps, as follows: Step 1 comprised validation of the implementation of the proposed deep learning model. All data of the pressed force and images of one pig kidney were trained, and the estimated forces from all identical images were used for training. The result was expected to be as close to 100% as possible. Step 2 comprised training the proposed model to achieve estimation within a sequence under the same conditions, using the data from the force measurements and images for one pig’s organs. In the experiment, 7148 total frames obtained from three videos were first divided into 80% training data and 20% test data. Early stopping was used to determine training convergence, with 10% of the training data used for validation. The average numbers of measurements in the training and test datasets used in the cross-validation were 1908 and 474, respectively. Step 3 comprised training the proposed model to estimate an unknown sequence not included in the training sequences. Seven of eight sequences under the same experimental conditions were used for training, and the proposed model estimated the force of the remaining sequence.

We used three pigs’ organs, and the estimation was performed for each pigs’ organs.

After confirming the difference in estimation accuracy from Steps 1–3, we evaluated the region of interest (ROI) of the proposed artificial intelligence (AI) model using a saliency map based on the results described below. As a result, we found that it is effective to restrict ROI as the cropping model instead of estimating the force from all the information in the surgical scenes as the non-cropping model to improve the estimation accuracy, and we describe the results below. An object detection model, the You Only Look Once (YOLO) method^13^, was used to detect the forceps tips. For more information, please refer to the Methods section.

### Error metrics

The error metrics were evaluated as follows: Forceps pressure $F$ as the force generated at the forceps tips was defined as the Euclidean norm of the measured values ($Fx, Fy, Fz$) of the three-axis forceps sensor, obtained using Eq. (1):

1 $$F = \sqrt { Fx^{2} + Fy^{2} + Fz^{2} } .$$

The measured ($f\_true$) and estimated ($f\_pred$) force generated at the forceps tip was then evaluated using two indices. Classification accuracy (CA) and mean absolute error (MAE) were set as evaluation indices for interpreting the results. CA set the threshold of safety manipulation for the force, and the precision of the estimations was validated using the ratio of the time frames during which forces exceeding and falling below 0.5 N were detected, as a metric. Here, N (Newton) is the force unit, and the threshold, 0.5N, was tentatively set on the basis of our research group’s previous research^14^. This research evaluated the degree of deformation when force is applied to organs and the magnitude of force generated during such deformation. The threshold was also set on the basis of discussions among surgeons of manipulations during the experiments. The MAE was used as an index to evaluate the accuracy of the estimation of the magnitude of the force generated by the comparison between the estimation and the measured value when the forceps pressed the organ. The value was calculated using Eq. (2), where m is the number of test data.

2 $$MAE = \frac{1}{m}\mathop \sum \limits_{i = 1}^{m} \left| {Fi - \hat{F}i} \right|.$$

In the comparison of the MAE values, it was expected that the values would be relatively larger with sequences involving high force vs low force. Therefore, to avoid these effects, each sequence was evaluated using the following error metric $E$ (Eq. 3), which compared the ratio of the absolute values of the difference between the measured and estimated values:

3 $$E = \frac{{\left| {\left( {f\_true - f\_pred} \right)} \right|}}{f\_true} \times 100.$$

This index was named the $\left| {\left( {f\_true - f\_pred} \right)} \right|/f\_true$ ratio.

### Evaluation

We evaluated the three steps as follows: In Step 1, when 100% of the data from the individual sequences were trained and forces were estimated from the identical video data, accurate prediction results were obtained for both CA and MAE, confirming that computationally, the model was working properly (Table 1). There was no difference in results with or without restricting the ROI.

**Table 1:** CA and MAE prediction results for force estimation from identical video data after 100% learning of each sequence’s data.

	P1		P2		P3	
	Non-cropping	Cropping	Non-cropping	Cropping	Non-cropping	Cropping
CA (%)	**98.6**	96.9	**99**	98.6	**98.6**	95.6
MAE (N)	**0.04**	0.108	**0.045**	0.083	**0.05**	0.134

Significant values are in [bold].

*P* Pig, *CA* Classification accuracy, *MAE* Mean absolute error.

In step 2, using 80% of the data for each sequence as training data, the remaining 20% of the data was used to estimate the forceps tips force. The results indicated that the accuracy of the estimation was maintained, even though the performance was worse than that in Step 1. The accuracy of the cropping model was better than that of the non-cropping model (Table 2).

**Table 2:** CA and MAE prediction results using 80% of each sequence’s data array for training and the remaining 20% for estimating forceps tips force.

	P1		P2		P3	
	Non-cropping	Cropping	Non-cropping	Cropping	Non-cropping	Cropping
CA (%)	69.1	**86.4**	80.6	**87.6**	75.9	**85.7**
MAE (N)	0.518	**0.274**	0.602	**0.406**	0.567	**0.328**

Significant values are in [bold].

*P* Pig, *CA* Classification accuracy, *MAE* Mean absolute error.

**Figure 2:**
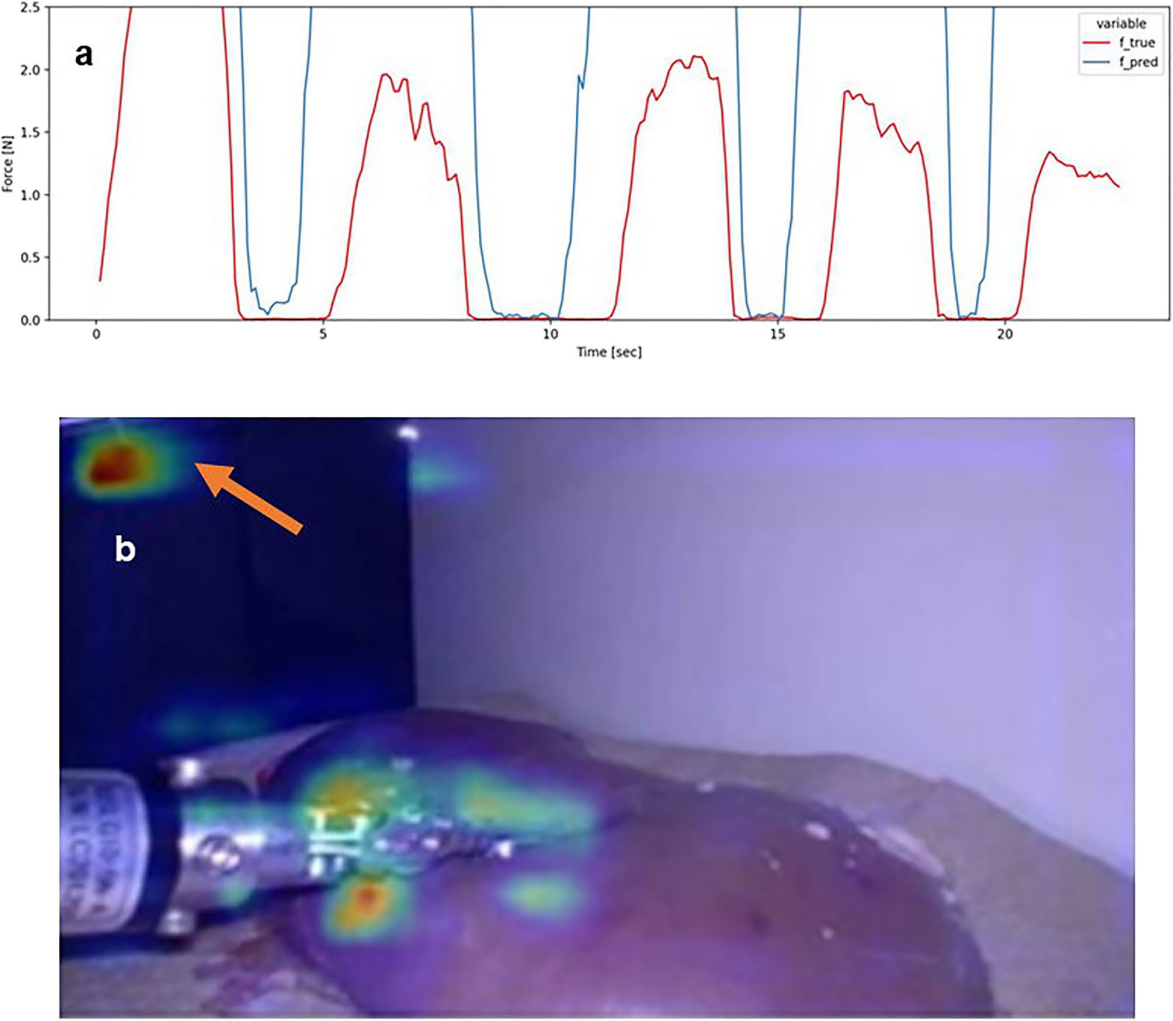
Forceps pressure estimation data in the non-cropping model. (**a**) Waveform of force estimation over time. () indicates the actual force generation and () indicates the force generation estimated by the model. Both the timing and magnitude of force generation are inaccurate. (**b**) The saliency map shows that the ROIs in the estimated model are distributed not only at the tips of the forceps but also in other parts of the surgical field image (). *ROI* Region of interest.

**Figure 3:**
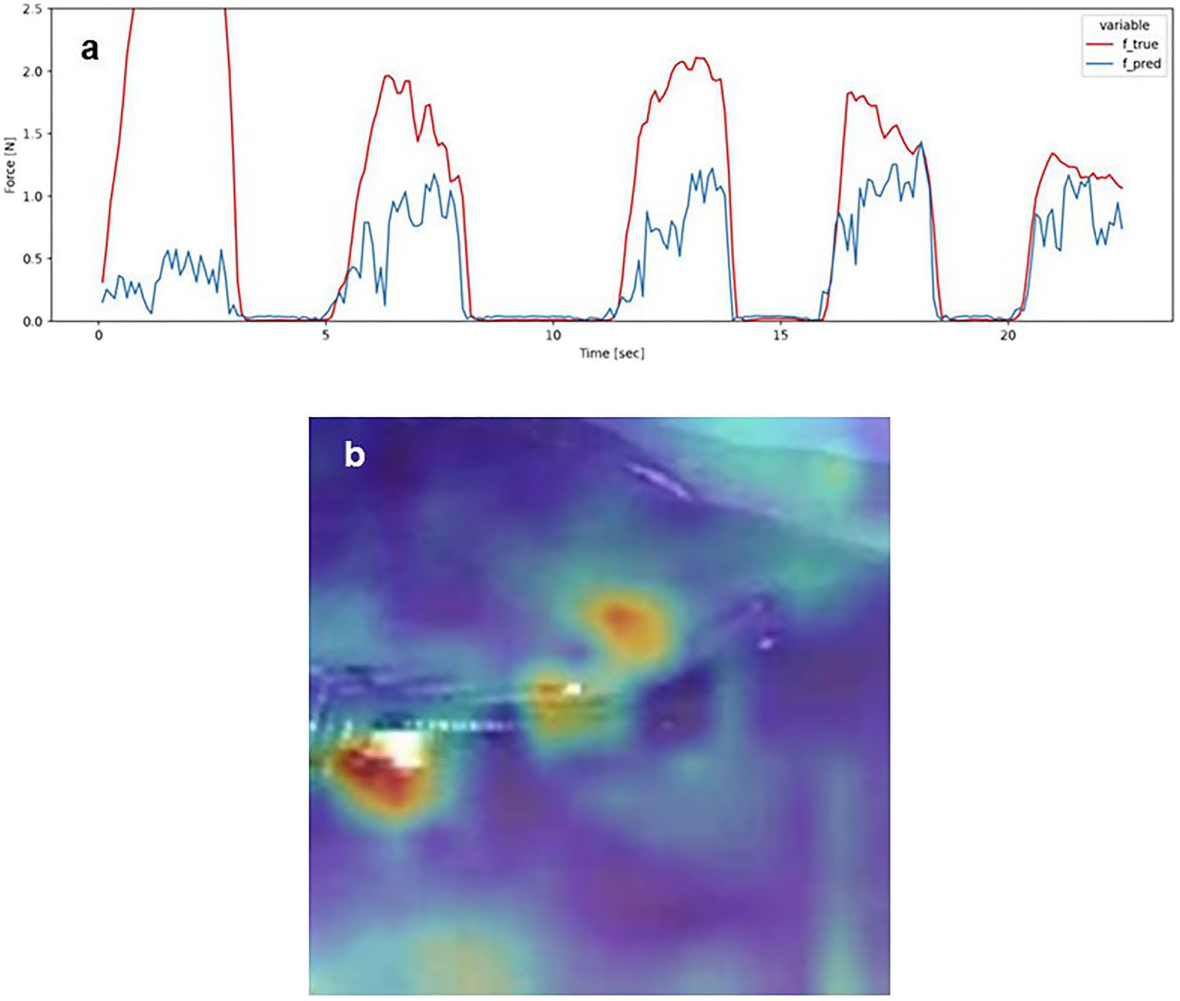
Forceps pressure estimation data in the cropping model. (**a**) Waveform of force estimation over time. () indicates the actual force generation and () indicates the force generation estimated by the model. Both the timing and magnitude of force generation are more consistent than those in the non-cropping model. (**b**) The saliency map shows that the ROIs in the estimated model are distributed at the tips of the forceps. *ROI* Region of interest.

**Figure 4:**
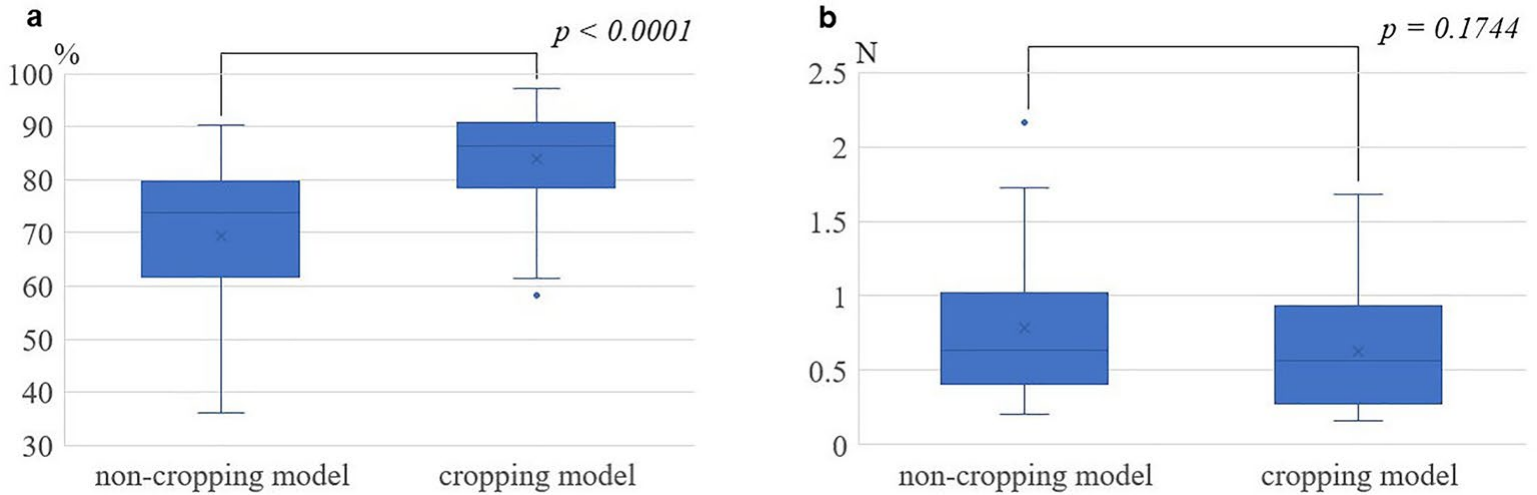
Comparison between non-cropping and cropping models. (**a**) Comparison of classification accuracy, with the cropping model having statistically significantly higher estimation accuracy than that with the non-cropping model. (**b**) Comparison of mean absolute error showing no statistically significant difference in estimation accuracy with either model.

Examples of the estimation results of the pressure on the forceps tips for a specific sequence in Step 3 are shown in Figs. 2 and 3. These figures show the data when the kidney of pig 2 was pressed, as follows: (i) more strongly than that in standard laparoscopic operations, (ii) in the X-axis direction, and (iii) in the middle of the kidney. Because the CA values were approximately median in all sequences, the conditions in Figs. 2 and 3 were selected from all the results. Figure 2 shows the analysis of the non-cropping model, and Fig. 3 shows the analysis of the cropping model. The estimated force waveforms ($f\_pred$) of the cropping model were more similar to the measured force waveforms ($f\_true$) than those of the non-cropping model. Figures 2b, 3b show the images of the saliency map. In the non-cropping model, the ROIs were dispersed, whereas in the cropping model, the ROI was concentrated around the forceps tips and the position where the organ was pressed (Figs. 2, 3). In fact, when each sequence for the three pigs was evaluated by the method described above and the CA and MAE were compared, no difference was observed for MAE. However, CA showed significantly better estimation accuracy in the cropping model compared with the non-cropping model (Fig. 4). The median CA was 73.7%/ 86.4% in the non-cropping/cropping models, respectively.

**Figure 5:**
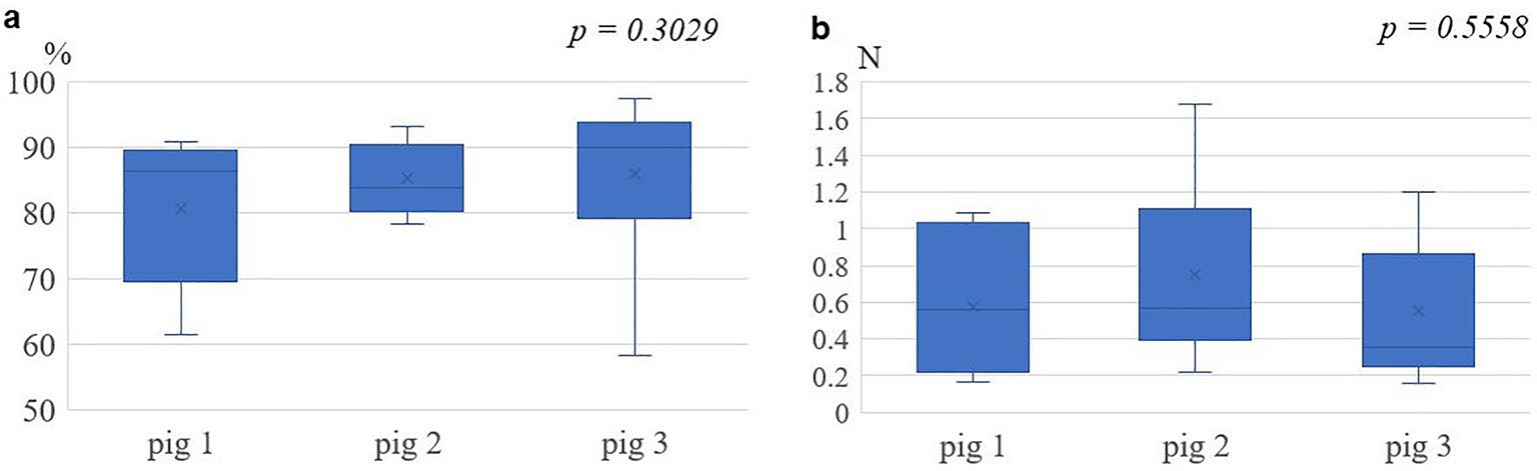
Estimation of accuracy among the three pig kidney experiments in the cropping model. (**a**) Comparison of classification accuracy. (**b**) Comparison of mean absolute error. There were no differences between the three experiments for either the classification accuracy or the mean absolute error.

Considering the effects of individual differences among the three pigs’ kidneys, the estimation accuracy of each of pig experiment was compared using the cropping model, and no significant difference was found for either CA or MAE (Fig. 5).

**Figure 6:**
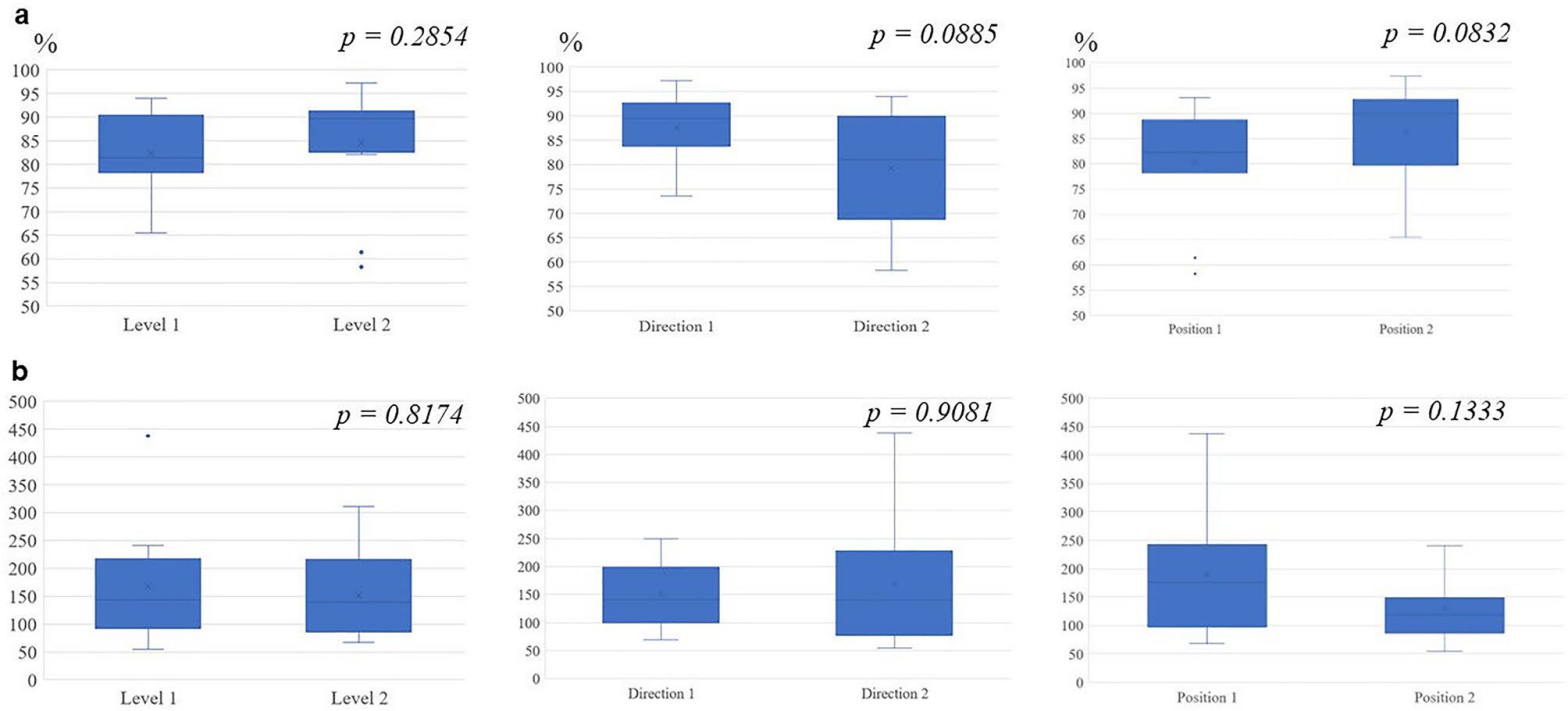
Comparison of power level, axial direction, and forceps position in the cropping model. (**a**) Comparison of classification accuracy. (**b**) Comparison of the $\left| {\left( {f\_true - f\_pred} \right)} \right|/f\_true$ ratio. Level 1: Force applied in normal laparoscopic surgery; Level 2: force above power level 1. Direction 1: X-axial compression; Direction 2: X + Z axial compression. Position 1: Middle of the pig kidney; Position 2: Edge end of the pig kidney.

Next, we examined whether the difference in accuracy between CA and MAE could be caused by the following factors in the cropping model: i) magnitude of the force of the forceps against the organ, ii) direction of the force, and iii) contact position. Considering the possibility of incorrect evaluation of the force magnitude by MAE, as described above, we used the $\left| {\left( {f\_true - f\_pred} \right)} \right|/f\_true$ ratio to evaluate the force magnitude. As shown in Fig. 6, there were no significant differences for each factor (Fig. 6). These results indicated that, to improve the accuracy of force estimation in monocular 2D endoscopic images, it is effective to restrict ROI (cropping model) instead of estimating the force from all information in the surgical scenes (non-cropping model).

Additionally, we confirmed the computational delay for vision-based force estimation. The average computation time for image cropping and forceps tip detection using YOLO was 16 ms, and the duration for CNN-based force prediction was 4 ms.

## Discussion

In this study, we developed and evaluated a simulation model using deep learning to estimate the force on forceps tips using only visual information real-time in surgery. We also evaluated the ROI of the AI model using a saliency map. To our knowledge, this is the first report showing that focusing on the forceps tips using the YOLO algorithm improves the accuracy of the estimation.

As surgeons who perform daily robotic surgery, we experience sensations similar to those of haptics in robot-assisted laparoscopic surgery, which should lack haptics. Additionally, during surgical instruction, the instructor makes judgments, including the strength of the force applied to organs, using only visual information. In both situations, these sensations are based on a surgeon’s actual clinical experience, and this study was inspired by the idea that the experience could be visualized.

The concept of “vision-based force sensing” has been studied in the field of biomedical engineering to determine the forces applied to organs based solely on recorded images^8–10^. However, as mentioned, many reports use systems that are not used in actual surgery. The uniqueness of this study is that the measurement environment of the forceps manipulation was carefully designed to match actual clinical practice, as follows: (1) We used an endoscopic system and monocular 2D endoscope that are used in clinical laparoscopic surgery. (2) The pressure measuring forceps were modified from actual surgical forceps. (3) The measured objects were not dummy models but excised organs. Additionally, the simulation model was designed to reflect the surgeon`s criteria, as follows: (1) The threshold danger zone for the applied force was defined, and whether the threshold could be estimated from the recorded images was evaluated using CA values. (2) Regarding the estimation by the AI, we evaluated the validity of the estimation process using a saliency map. (3) Our results showed that it is effective to restrict ROI (cropping model) rather than to estimate the force from all information in the surgical scenes (non-cropping model) to improve the accuracy of the force estimation.

All surgical scene images (non-cropping model) and the ROI-restricted images (cropping model) were prepared for the visual information to determine the ideal condition for AI estimation. The average computation time for the cropping model was 20 ms. In terms of human visual characteristics, this means that real-time notification of predicted results is possible by both cropping and non-cropping models and can be used as intraoperative anomaly detection for excessive pressure. However, further psychological study is needed to determine how this delay affects the human senses for the purpose of tactile feedback.

In this study, we focused on CA as well as MAE, based on the force unit, which is the magnitude of the actual force, for the forceps tips pressure estimation. As a result of this investigation, while the estimation accuracy based on MAE is currently considered insufficient, we believe that the potential for determining whether the applied force exceeds or falls below a predefined threshold has been demonstrated through the results based on CA. However, we do not prioritize results based on CA over those based on MAE, and intend to perform further research in this area. We believe that improvements in the model, such as incorporating prior knowledge of tissue deformation, could lead to more accurate estimations. Notably, on the basis of experience with pseudo-haptic feedback, there is room for exploration regarding the extent of the clinical need to precisely quantify and represent everything as tactile sensations. Additionally, creating a surgical robot that is capable of representing haptics requires structural modifications. Therefore, a haptics representation technique implemented on a surgical robot is limited in application to other operations, such as laparoscopic surgery and open surgery.

Robot-assisted surgery lacks haptics, and the presence or absence of force feedback significantly affects surgical performance in novice surgeons, with less of an effect in experienced surgeons^15^. In this study, we judged that alerting the surgeon when the force exceeds a certain level by setting the threshold of the safety zone is more important than clearly indicating the detailed force in real-time. However, we believe that improving the estimation to provide some level of detail regarding the magnitude of the force will further enhance the accuracy of safety zone determinations. This concept is similar to that in devices that reduce the risk of accidents due to human error, such as automatic braking devices and devices that prevent the mistake of incorrectly stepping on the brake or accelerator pedal. Although such devices have been developed and implemented in other medical fields, no human error prevention devices have been developed and implemented to the same extent in surgical devices. Human error can occur not only for novice surgeons but also for experienced surgeons. As a possible aid, superimposing saliency maps onto intraoperative camera images in real-time could be useful. It is possible to manage dangerous surgical operations by robot-assisted surgery compared with non-robotic surgery, including open surgery. We believe that this study represents an important step forward in the development of a safety control device for surgeons in robotic surgery.

The median MAE value in this study exceeded 0.5 N for both the non-cropping model and the cropping model (Fig. 4b). The current model has limitations in estimating the magnitude of the force in detail in real-time using a monocular camera. To improve accuracy, a binocular 3D endoscopic system instead of a monocular 2D endoscope is a consideration. However, the required amount of information processing using a 3D system is very large; therefore, image processing techniques must be considered. Another approach to enhancing accuracy comprises expanding the training dataset, diversifying the conditions under which data is captured for deep learning purposes, and incorporating semi-supervised learning techniques, all of which are promising.

The limitations of this study are as follows: (1) This study was not performed on a living organ, (2) we performed a limited operation using forceps to apply pressure to organs, and (3) the endoscope was fixed, to obtain steady images. In the present study using a box trainer for surgical practice, the surroundings were inorganic structures except for the excised kidneys. However, in actual surgery, many surrounding organs are affected. Although the accuracy of the model may be lower than that in actual surgery because of the model design, we expect that restricting the ROI to the forceps tips may result in good performance in the complicated environment of actual surgery. Additionally, in actual surgery, it may be more difficult to fix the images owing to the dynamic nature, compared with a box trainer. Further study is needed to overcome this problem.

## Methods

To perform this study, we considered the following: (i) the measurement data of the magnitude of force generated at the forceps tips (forceps pressure) over time when organs were pressed with forceps, and (ii) the recording data of the images of organ deformation over time. For (i), we performed item (1), below, and for (ii), we performed item (2), below. The relationship between (i) and (ii) was consistent with the time series.

1. Creation and measurement of the forceps force with force sensors. In this study, forceps that can measure the magnitude of the force generated at the forceps tips when an organ is pressed were necessary. To simulate actual clinical practice, we developed a forceps-equipped three-axis sensor (USLG10-5N: Tec Gihan Co., Ltd.) based on the forceps tip of a surgical robot (Fig. 1a). We considered that the sensor position was very important for the accuracy of the data. Previous studies have positioned the sensor at the base of the forceps, a location considerably distant from the forceps tips^10^, or the sensor sheet was placed behind the organ to be pressed^16^. However, organs are elastic, and the measurement value may be attenuated when the measurement position is far from the point of action. Therefore, we decided that it was appropriate to place the force gauge as close as possible to where the force is generated, for accuracy. Additionally, the forceps were designed with the assumption that organs are pressed under the same conditions as those in actual surgery. The rated capacity of the force sensors was ± 5 N, and the forces ($Fx, Fy, Fz$) in each axial direction could be measured at a refresh rate of 100 Hz. Figure 1 shows each axial direction, where the Z-axis means that the force is applied in the vertical direction regarding the forceps. To simplify the analysis, the forceps pressure to be estimated was assumed to be a value independent of the forceps posture and axial rotation. That is, the forceps pressure $F$ was defined as the Euclidean norm of the triaxial forceps sensor measurements obtained using Eq. (1):
2. Creating a deep learning model for forceps pressure estimation—collection of basic data

To create a deep learning model under conditions similar to those of actual surgery, we recorded images using a monocular 2D endoscope. Excised pig kidneys were used as the surgical organ. We performed similar manipulations on the excised kidneys, assuming that the kidney was pressed and deformed during surgery. To obtain training data that reflect variations in the pressing operation, we performed multiple trials of pressing on the kidneys under different conditions. The camera position was fixed, to obtain steady images. The basic data were collected by this method (see the Results section).

In the data processing and model creation, synchronization was performed on the independently measured forceps pressure data and the endoscopic images. To confirm the baseline estimation performance, a CNN regression model that accepts a single 2D endoscopic image “$I$” as the input image and outputs a 3D stress vector ($Fx, Fy, Fz$) was used as the deep learning model. This CNN was composed of 13 layers based on the VGG-16 architecture. The 13 layers were as follows: a convolution layer consisting of convolution, batch normalization, and max pooling, and a total concatenation layer. The convolution layer included a dropout to prevent overlearning. In this study, the obtained force vector was converted to a scalar value of forceps pressure using Eq. (4), and the value was used as the predicted value. The network was optimized using the mean squared error defined by the equation as the loss function $L$, where $Fi$ is the true forceps pressure measurement, $\hat{F}i$ is the prediction, and $n$ is number of training data.

4 $$L = \frac{1}{n}\mathop \sum \limits_{i = 1}^{n} \left( {Fi - \hat{F}i} \right)^{2}$$

To provide a visual understanding of the regions used for prediction in the input image and the image features, the gradient of the output of the lowest convolutional layer was calculated based on the saliency map, and the gradient map was overlaid on the input image for visualization.

The CNN was implemented using the Keras library with Python 3.9 and Tensorflow 2.6, as the backend. The batch size was set to 60 and the training epoch to 1000, and the network was optimized using Adam with a learning rate of $1 \times 10^{ - 4}$. The movie frame was resized to $640 \times 360$ pixels and used as an input for the non-cropping model. The cropping area for the cropping model was set to $128 \times 128$ pixels and obtained from the resized movie frame.

### Model novelty: set the ROI of the AI model

We set the ROI of the AI model as (1) the entire scene of the surgical field (non-cropping model) or (2) the tips of the forceps (cropping model), and examined whether there was a difference in the accuracy of the estimation. To adjust the ROI, we focused on the structures near the forceps tips and used the YOLO method for recognition by deep learning.

### Evaluation method

We used the data with various differences of conditions as sequences, and the MAE was used in the MAE comparison with the measured values in the estimation experiment. The MAE was calculated using Eq. (2), where m is the number of test data:

The danger zone was set for the force of the forceps pressed on the kidneys, and the ratio of the time when a force of ≥ 0.5 N could be judged and when a force of ≤ 0.5 N could be judged was calculated and evaluated as CA. The conditions that make force estimation difficult and methods to improve the accuracy of the force estimation were examined using a combination of sequences. Additionally, to enable a visual understanding of the regions and image features used for prediction in the input images, a saliency map was created from the gradient of CNN weights and superimposed onto the input images.

### Statistical analysis

In each experimental condition, we computed the CA and MAE values. These were subsequently compared utilizing the Wilcoxon rank-sum test and the Kruskal–Wallis test. We considered *p* < 0.05 statistically significant. All statistical analyses were performed using JMP^®^ Pro 17.0.0 (SAS Institute Inc.).

## Conclusion

Pseudo-haptic sensations are experienced in surgery by skilled surgeons. We aimed to provide a visualization method representing actual force on the manipulation point of forceps using an AI model. The efficiency of the force estimation between the different ROIs was evaluated. A sensor forceps was developed, and the actual force on excised organs was measured with the manipulation images. Using the YOLO method to detect the tips of the forceps and developing a new method to limit the ROI in AI, the median CA in the non-cropping/cropping models, which was the evaluation method in Step 3, was significantly better than that without limiting the ROI. The median CA was 73.7%/86.4% in the non-cropping/cropping models, respectively. We concluded that the restricted ROI (cropping model) is effective to improve the estimation. These findings indicate that the proposed simulation model would be appropriate for use in complicated environments in actual surgery, in the future.

## Data Availability

The datasets used and/or analyzed during the current study are available from the corresponding author on reasonable request.
